# Bacteriophage-encoded virion-associated enzymes to overcome the carbohydrate barriers during the infection process

**DOI:** 10.1007/s00253-017-8224-6

**Published:** 2017-03-23

**Authors:** Agnieszka Latka, Barbara Maciejewska, Grazyna Majkowska-Skrobek, Yves Briers, Zuzanna Drulis-Kawa

**Affiliations:** 1 Department of Pathogen Biology and Immunology, Institute of Genetics and Microbiology, University of Wroclaw, Przybyszewskiego 63/77, 51-148 Wroclaw, Poland; 2 Laboratory of Applied Biotechnology, Department of Applied Biosciences, Ghent University, Valentin Vaerwyckweg 1, 9000 Ghent, Belgium

**Keywords:** Bacteriophages, Polysaccharide depolymerases, Virion-associated lysins

## Abstract

Bacteriophages are bacterial viruses that infect the host after successful receptor recognition and adsorption to the cell surface. The irreversible adherence followed by genome material ejection into host cell cytoplasm must be preceded by the passage of diverse carbohydrate barriers such as capsule polysaccharides (CPSs), O-polysaccharide chains of lipopolysaccharide (LPS) molecules, extracellular polysaccharides (EPSs) forming biofilm matrix, and peptidoglycan (PG) layers. For that purpose, bacteriophages are equipped with various virion-associated carbohydrate active enzymes, termed polysaccharide depolymerases and lysins, that recognize, bind, and degrade the polysaccharide compounds. We discuss the existing diversity in structural locations, variable architectures, enzymatic specificities, and evolutionary aspects of polysaccharide depolymerases and virion-associated lysins (VALs) and illustrate how these aspects can correlate with the host spectrum. In addition, we present methods that can be used for activity determination and the application potential of these enzymes as antibacterials, antivirulence agents, and diagnostic tools.

## Introduction

Bacteriophages (phages) are bacterial predators. Every phage infection is initiated by adsorption (i.e., the attachment of the phage particle to the bacterial host cell), which is characterized by the specific recognition of a receptor located on the bacterial cell surface. Subsequently, phages have to penetrate the bacterial cell envelope and will eventually eject their viral genome into the cytoplasm (Weinbauer 2004). On the way to the bacterial cytoplasm, phages have to overcome different carbohydrate barriers. Some bacteria produce capsular polysaccharides (CPSs; also called K-antigens), which are tightly attached to the cell. Other bacteria secrete slime with extracellular polysaccharides (EPSs) to the environment during biofilm formation (Schmid et al. 2015). Both CPS and EPS have a huge diversity in composition, not only within a bacterial genus but also within a species, and this composition can also vary dependent on the growth phase and environmental conditions (Corbett and Roberts 2008; Leiman and Molineux 2008). Gram-negative bacteria are characterized by an outer membrane composed of lipopolysaccharide (LPS) molecules with a highly variable O-polysaccharide (O-antigen), whereas Gram-positive bacteria have essential components such as (lipo)teichoic acids embedded in peptidoglycan (PG). These polysaccharides are often a primary receptor, along with other protruding cell wall structures such as pili and flagella. Bacteriophages have evolved various virion-associated carbohydrate active enzymes, termed polysaccharide depolymerases, that recognize, bind, and degrade the polysaccharide compounds to gain access to the initially inaccessible bacterial cell surface receptor (Leiman et al. 2007; Leiman and Molineux 2008; Li et al. 2016). This secondary receptor (co-receptor) can be an outer membrane protein, a cell wall embedded protein (structural proteins, transport channels, enzymatic and secreting proteins), or a distal part of the exopolysaccharides (Nilsson et al. 2000; Bertozzi Silva et al. 2016). Binding of this receptor will eventually trigger DNA ejection (Leiman and Molineux 2008). To transfer the genome to the bacterial cytoplasm, bacteriophages have to overcome another rigid carbohydrate barrier, i.e., the PG. This structure comprises up to 40 layers in case of Gram-positive bacteria and 1–3 layers in case of Gram-negative bacteria. Whereas the chemotype of the PG of Gram-negative bacteria is conserved (A1γ), the PG composition of Gram-positive bacteria varies significantly in peptide composition, cross-link, and modification of the glycan chain (Briers et al. 2007b). Therefore, phage particles are equipped with a virion-associated lysin. In contrast to the lysis phase where PG is extensively degraded by endolysins, the virion-associated lysins only locally puncture a hole in the PG, which is sufficiently large to eject the phage DNA into the cytoplasm.

This review gives an overview about the recent knowledge of virion-associated carbohydrate active enzymes. Whereas virion-associated lysins are a prerequisite for every phage as PG is an inevitable barrier, polysaccharide depolymerases provide the phages with tools to have a competitive advantage in niches (e.g., in biofilms, these enzymes allow to gain access to the microcolonies and to infect embedded bacteria (Abedon 2012) and to differentiate in host spectrum). The study on LPS-degrading depolymerase suggested that the low enzyme kinetics may be correlated with O-chain degradation important for detachment of released progeny, rather than for initial adsorption step (Steinbacher et al. 1996). Both groups of carbohydrate active enzyme share common features such as a customized enzyme specificity evolved throughout a continuous arm race between phage and host and their structural location. Some reviews dealing with these phage-encoded, virion-associated enzymes have been published recently, particularly focusing on general characteristics and biomedical applications (Drulis-Kawa et al. 2012, 2015; Yan et al. 2014) or on virion-associated peptidoglycan hydrolases (VAPGHs) (Rodríguez-Rubio et al. 2013a). Pires et al. (2016) recently provided an overview of 160 putative depolymerases in 143 phages infecting 24 genera of bacteria based on an extensive in silico analysis. Here, we present a summary of experimentally confirmed polysaccharide depolymerases and virion-associated lysins (VALs) and focus on the main characteristics of the corresponding genes, their protein structure, their location within the virion particle, their mode of action and specificity, evolutionary aspects, methods for activity determination, and potential applications.

## Polysaccharide depolymerases

The hallmark feature for the presence of phage-encoded polysaccharide depolymerases was firstly described in 1956 as the plaque-surrounding halos, which increase in diameter over time of incubation while the plaque size remains constant. The depolymerases responsible for the degradation of CPS, EPS, or O-polysaccharide can be virion-associated as an integral part of the virion particle or can be in a soluble form, being released during bacterial cell lysis without being integrated in a phage particle (Adams and Park 1956; Stirm et al. 1971). Both can freely diffuse and cause a time-dependent polysaccharide degradation resulting in the typical halo-like appearance around plaques. In spite of polysaccharide degradation, phages do not establish an infection since the bacterial cells in the halo are not actively propagating.

### Enzymatic activity and specificity of polysaccharide depolymerases

Bacteriophage-encoded depolymerases can be divided into two main classes according to their mechanism of action, i.e., hydrolases (EC 3) and lyases (EC 4), both resulting in the cleavage of polysaccharides into soluble oligosaccharides and the breakdown of the carbohydrate barrier. Within both classes, there is a tremendous variation in specificity responding to the large existing diversity in bacterial polysaccharides (CPS, EPS, O-polysaccharides). This high specificity usually contributes to the narrow host spectrum. The majority of hydrolases belong to the group of O-glycosyl hydrolases (EC 3.2.1), which use a water molecule to cleave specifically the O-glycosidic bonds of the polysaccharide. This group comprises sialidases, rhamnosidases, levanases, xylanases, and dextranases (Davies and Henrissat 1995; Pires et al. 2016). Polysaccharide depolymerases for which experimental data are available are mainly derived from phages propagating on the most common human pathogens (Table 1).

**Table 1:** Phage-encoded polysaccharide depolymerases

Host	Phage phylogeny	Phage	Protein name/accession code	Monomer mass (kDa)/oligomeric state (if known)	Activity	References
Capsule-degrading depolymerases						
*Escherichia coli*	*Podoviridae*	K1A	ABP02011.1	90/trimer	Endo-N-acetylneuraminidases	Jakobsson et al. (2007)
		K1E	CAA85449.2	90/trimer	Endo-N-acetylneuraminidases	Tomlinson and Taylor (1985), Gerardy-Schahn et al. (1995), and Long et al. (1995)
		K1F	AAZ73001.1	119/trimer	Endo-N-acetylneuraminidases	Hallenbeck et al. (1987), Petter and Vimr (1993), Muhlenhoff et al. (2003), and Stummeyer et al. (2005)
		Φ1.2	ND	ND	Endo-N-acetylneuraminidases	Kwiatkowski et al. (1983)
		K1–5	ORF46 AAG59821.1 ORF47 AAG59822.1	67/mature form 52/trimer 90/trimer	poly(β-1,4-GlcA-α-1,4-GlcNAc) lyase Endo-N-acetylneuraminidases	Scholl et al. (2001) and Scholl et al. (2004)
		K5A	KflA Y10025.2	67/trimer	poly[-4)-β GlcA-(1,4)-α GlcNAc-(1-] lyase	Clarke et al. (2000) and Thompson et al. (2010)
		CUS-3	CAJ29292.1	109	Endo-N-acetylneuraminidases	Stummeyer et al. (2006)
		29	ND	57/trimer^a^	Glycanase	Rieger et al. (1975)
		K30	ND	142^a^	Glycanase	McCallum et al. (1989)
	*Siphoviridae*	63D	ADA82273.1	108/tetramer	Endo-N-acetylneuraminidases	Machida et al. (2000)
	*Myoviridae*	*Φ*92	Gp143 CBY99572.1 Gp141 CBY99570.1 Gp142 CBY99571.1 Gp150 CBY99579.1 Gp151 CBY99580.1	102	Endo-N-acetylneuraminidases Glycosidase Glycosidase Colonidase tailspike Potential tail fiber	Schwarzer et al. (2012)
*Klebsiella pneumoniae*	*Podoviridae*	11	ND	63^a^	Hydrolase	Thurow et al. (1974) and Bessler et al. (1973)
		KPO1K2	ND	36^a^	Hydrolase	Kassa and Chhibber (2012)
		NTUH-K2044-K1–1	K1-ORF34 YP_009098385.1	70	Pectate lyase with polygalacturonase domain	Lin et al. (2014)
		P13	Genes 49 and 50	62–65^a^	Putative hydrolase	Liu et al. (2014) and Shang et al. (2015)
		20	ND	ND	Galactosidase	Thurow et al. (1974)
		24	ND	ND	Glucosidase	Thurow et al. (1974)
	*Siphoviridae*	KP36	Gp50 YP_009226011.1	94/trimer	Putative lyase	Majkowska-Skrobek et al. (2016)
		KLPN1	ORF34 YP_009195374.1 and/or ORF35 YP_009195375.1	26 24	Endo-N-acetylneuraminidases	Hoyles et al. (2015)
	*Myoviridae*	0507-KN2–1	ORF96 YP_008532047.1	134	Endo-N-acetylneuraminidases	Hsu et al. (2013)
		ΦK64–1	S1–1 BAQ02837.1 S1–2 BAQ02835.1 S1–3 BAQ02836.1 S2–1 BAQ02838.1 S2–2 BAQ02839.1 S2–3 BAQ02840.1 S2–4 BAQ02841.1 S2–5 BAQ02842.1 S2–6 BAQ02843.1 S2–7 BAQ02844.1 S2–8 BAQ02805.1	76 82 71 131 62 86a 97 110 83 74 63	Tail fiber Tail spike Tail spike Tail fiber Tail fiber Lyase Tail fiber Tail fiber Pectate lyase Tail fiber Tail fiber	Pan et al. (2017) and Pan et al. (2015)
*Pseudomonas* sp.	*Podoviridae*	Φ15	Gp17 YP_004286222.1	78	Pectin lyase	Cornelissen et al. (2011)
		PT 6	ND	37^a^	Alginate lyase	Glonti et al. (2010)
		F116	YP_164318.1	94	Putative glycosyl hydrolase	Hanlon et al. (2001)
		AF	gp19 YP_007237194.1	79/trimer	Pectin lyase	Cornelissen et al. (2012)
* Azotobacter vinelandii*		ND	ND	30–35^a^	Alginate lyase	Davidson et al. (1977)
*Vibrio cholerae*	*Podoviridae*	JA1	YP_008126828.1	87	Lyase	Linnerborg et al. (2001)
LPS-degrading depolymerases						
*Escherichia coli*	*Podoviridae*	HK620	NP_112090.1	77/trimer	Endo-N-acetylglucosaminidase	Barbirz et al. (2008)
	ND	Omega8	ND	35^a^	Endo-α-1,3-mannosidase	Prehm and Jann (1976)
* Salmonella enterica*	*Podoviridae*	P27	ND	ND	Endorhamnosidase	Wollin et al. (1981)
		KB1	ND	ND	Endorhamnosidase	Wollin et al. (1981)
		P22	Gp9 NP_059644.1	72/trimer	Endorhamnosidase	Iwashita and Kanegasaki (1976a), Baxa et al. (1996), and Steinbacher et al. (1996, 1997)
		SP6	ORF46 NP_853609.1 ORF47 NP_853610.1	59 53	Endorhamnosidase Putative endopolygalacuronase	Scholl et al. (2002) and Scholl et al. (2004)
	*Siphoviridae*	9NA	YP_009101225.1	73	Endorhamnosidase	Wollin et al. (1981) and Casjens et al. (2014)
	*Myoviridae*	Det7	CAO78738.1	75/trimer	Endorhamnosidase	Walter et al. (2008)
* Salmonella anatum*	*Podoviridae*	Epsilon15	Gp20 NP_848228.1	116/trimer	Endorhamnosidase	Chang et al. (2010) and Guichard et al. (2013)
		C341	ND	ND	Deacetylase	Iwashita and Kanegasaki (1976b)
*Shigella flexneri*	*Podoviridae*	Sf6	AAD33394.2	67	Endorhamnosidase	Chua et al. (1999) and Freiberg et al. (2003)
Depolymerases from Gram-positive infecting phages						
*Streptococcus pyogenes*	Prophage	370.1	hylP1 AAK33657.1	36/trimer	Hyaluronidase	Baker et al. (2002) and Smith et al. (2005)
		370.2	hylP2 AAK33900.1	40/trimer	Hyaluronidase	Hynes et al. (1995) and Mishra et al. (2006)
		370.3	hylP3 AAK34249.1	40	Hyaluronidase	Martinez-Fleites et al. (2009)
*Streptococcus equi*	Prophage		WP_015898604 YP_002747260	40	Hyaluronidase	Lindsay et al. (2009)
*Staphylococcus* sp.	*Siphoviridae*	ΦIPLA7	Dpo7 orf 18	98	Pectate lyase	Gutierrez et al. (2015)

*ND* no data available

^a^No AA sequence available; molecular mass was determined by other techniques

Probably, the best studied depolymerases are the sialidases or endo-N-acetylneuraminidases originating from *Escherichia coli* K1 specific bacteriophages such as K1A, K1E, K1F, K1–5, 63D, CUS-3, Φ1.2, and Φ92 (Kwiatkowski et al. 1983; Petter and Vimr 1993; Gerardy-Schahn et al. 1995; Long et al. 1995; Machida et al. 2000; Scholl et al. 2001; Muhlenhoff et al. 2003; Stummeyer et al. 2005; Stummeyer et al. 2006; Jakobsson et al. 2007; Schwarzer et al. 2012). Sialidases hydrolyze internal α-2,8-linkages in capsular polysialic acid. Proteins possessing endosialidase domains have also been found in *Klebsiella* phages KLPN1 and 0507-KN2–1 (Hsu et al. 2013; Hoyles et al. 2015) (Table 1). Rhamnosidases are enzymes able to cleave the α-1,3 O-glycosidic bond between L-rhamnose and D-galactose present in the O-antigen of *Salmonella* LPS. They have been described as tailspikes in phages specific for *Salmonella* (P27, KB1, P22, 9NA, Det7, Epsilon15) and *Shigella* (Sf6) (Wollin et al. 1981; Steinbacher et al. 1996, 1997; Baxa et al. 1996; Chua et al. 1999; Freiberg et al. 2003; Walter et al. 2008; Chang et al. 2010; Guichard et al. 2013). *E. coli* LPS-degrading enzymes were found as endo-N-acetylglucosaminidase and endo-α-1,3-mannosidase in HK620 and Omega8 phages, respectively (Barbirz et al. 2008; Prehm and Jann 1976) (Table 1). A levanase catalyzes hydrolysis of the β-2,6-bond between fructose monomers in levan, which is present in *Bacillus* biofilms and has been identified in *Bacillus* phage SP10. A xylanase responsible for hydrolysis of the β-1,4 bonds within xylan has been identified in the *Caulobacter* phage Cr30, while a dextranase cleaving the α-1,6-linkages between glucose units in dextran is predicted in *Lactobacillus* phage ΦPYB5 (Pires et al. 2016). Some virion-associated depolymerases do not depolymerize polysaccharides but cleave polypeptides (EC 3.4) or lipids (EC 3.1), responding to the different nature of some bacterial capsules. A peptidase enzyme digesting the poly-γ-glutamate capsular polypeptide of *Bacillus* sp. has been experimentally verified for *Bacillus* phage ΦNIT1 (Kimura and Itoh 2003). Pires et al. (2016) predicted also virion-associated lipases (triacylglycerol hydrolases) in eight *Cellulophaga* phages and one *Pseudomonas* phage, but experimental validation is currently lacking. A last group of virion-associated depolymerases with hydrolytic activity are LPS deacetylases in the tailspikes of the Vi phages (II and III) as well as those from *Salmonella anatum* phage c341, which deacetylate the O-antigen rather than breaking the polysaccharide chain (Iwashita and Kanegasaki 1976b).

Other polysaccharide depolymerases belong to the class of lyases, which differentiate from hydrolases as they cleave a glycosidic bond by β-elimination with the concomitant introduction of new double bond and without the use of a water molecule. Precisely, this group covers widespread hyaluronate, pectate/pectin, and alginate lyases and the specific K5 lyase (Sutherland 1995). Bacteriophage-encoded hyaluronidases (hyaluronate lyases) cleave the β-1,4 bonds between the subunits of hyaluronic acid and were found in prophages invading *Streptococcus pyogenes* and *S. equi*, which are both encapsulated by hyaluronic acid (Hynes et al. 1995; Ferretti et al. 2001; Baker et al. 2002; Smith et al. 2005; Mishra et al. 2006; Martinez-Fleites et al. 2009; Lindsay et al. 2009) (Table 1). Those enzymes are also used by the lysogenic host bacterium as a virulence factor to invade and penetrate human tissues. Pectate/pectin lyases are characterized by cleavage of the α-1,4 bonds of polygalacturonic acid. Enzymes possessing such domain have been described for *Pseudomonas* phages Φ15 and AF (Cornelissen et al. 2011, 2012), *Klebsiella* phages (NTUH-K2044-K1–1, KP36) (Lin et al. 2014; Majkowska-Skrobek et al. 2016), *Vibrio* phage JA1 (Linnerborg et al. 2001), and *Staphylococcus* phage vB_SepiS-ΦIPLA7 (Gutierrez et al. 2015) (Table 1). Alginate lyases (mannuronate or guluronate lyases) characteristic for *Pseudomonas* and *Azobacter* phages (PT6) are able to degrade the α-1,4 bond of alginate, a linear polysaccharide of β-D-mannuronate, and its C5 epimer α-L-guluronate common for mucoid strains infecting cystic fibrosis patients (Davidson et al. 1977; Wong et al. 2000; Glonti et al. 2010) (Table 1). The *E. coli* K5 capsular polysaccharide poly(β-1,4-GlcA-α-1,4-GlcNAc) is a receptor of the capsule-specific bacteriophages K5A and K1–5 that both produce a specific lyase acting on the α-1,4 bond (Clarke et al. 2000; Thompson et al. 2010).

### Conserved structural features of polysaccharide depolymerases

**Fig. 1:**
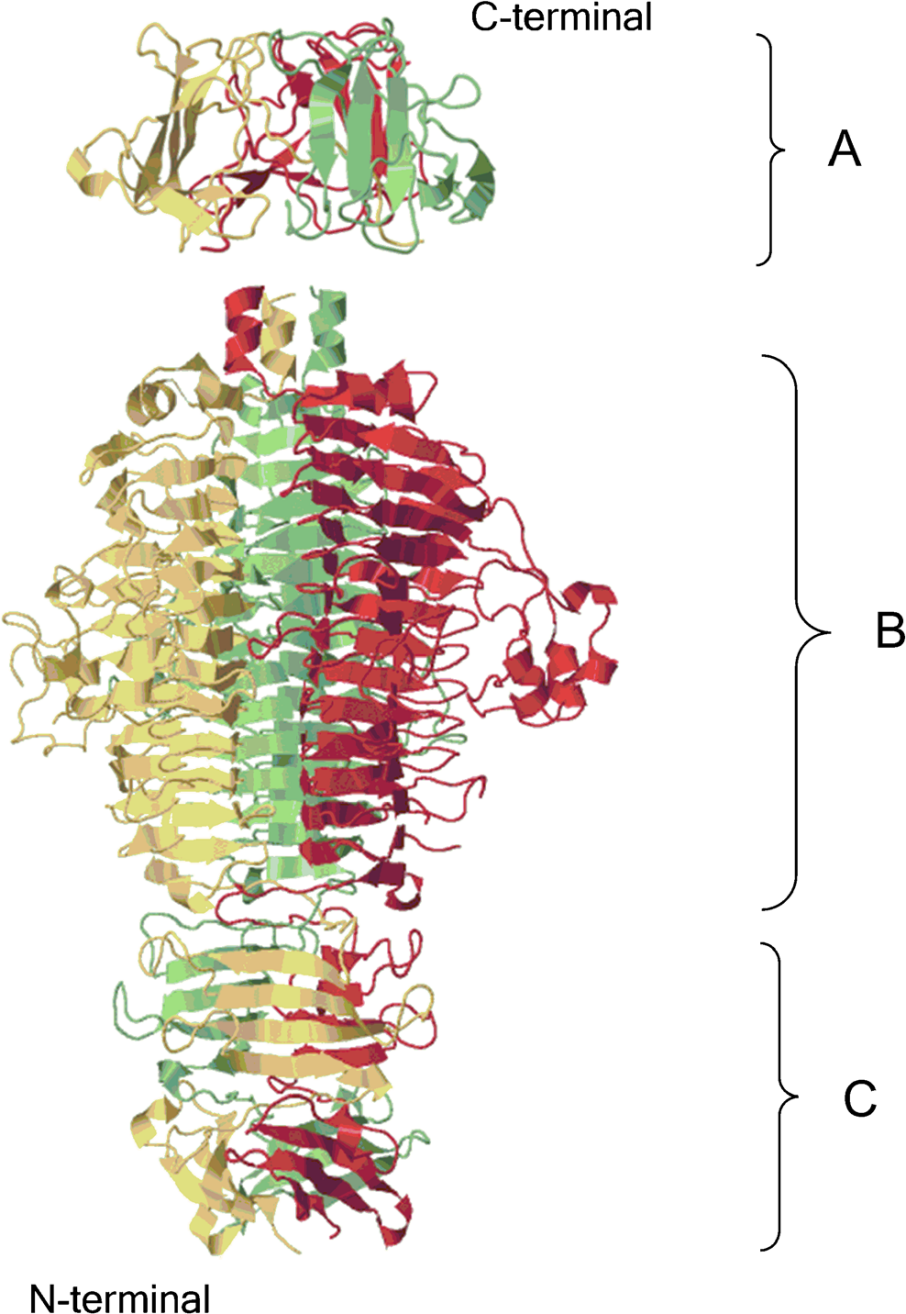
Structural architecture of phage tailspike/depolymerase on the base of P22 tailspike (PDB ID 1LKT and 1TYV; JavaScript Protein Viewer). N-terminal dome-like structure domain (**a**). Central domain for host recognition and enzymatic activity (**b**). C-terminal domain responsible for protein trimerization or/and receptor recognition (**c**)

In spite of the large diversity in enzymatic specificity, depolymerases share strikingly common structural features. Depolymerases described and characterized to date are present in the phage virion within tail fibers or tailspikes on the baseplate, regardless of the phage family they originate from or the host bacterial species they infect (Table 1). One described exception is the depolymerase encoded by *Staphylococcus* phage vB_SepiS-ΦIPLA7, which shows 99% homology with a pre-neck appendage protein (Gutierrez et al. 2015). Generally, depolymerases form elongated homotrimers that appear as protruding cell-puncturing devices on the virion, which aligns well with their biological function. The endosialidase encoded by *E. coli* siphophage 63D, however, was characterized as a homotetramer (Machida et al. 2000). On the structural level, depolymerases are fibrous proteins with a parallel β-helix topology composed of parallel β-strands orthogonal to the long axis (Fig. 1). This protruding shape extends the active site to recognize and bind specific sequences buried within cell surface polysaccharides (Weigele et al. 2003). The P22 tailspike with rhamnosidase activity degrading the LPS O-polysaccharide of *Salmonella typhimurium* binds its substrate along its entire length (Steinbacher et al. 1996). Therefore, the protruding shape of phage-encoded polysaccharide depolymerases has been proposed to create a long lateral surface for reading the specific polysaccharide sequence, determining the host specificity (Bradley et al. 2001). The complex structure of highly interwoven β-sheets also determines the high stability of these proteins, since they were found to be resistant to high temperature (possessing relatively high melting point temperature), proteases, and detergents at room temperature. This high stability corresponds to the harsh external conditions these proteins have to withstand in different environments such as the presence of proteases and denaturing conditions (Yan et al. 2014; Majkowska-Skrobek et al. 2016).

### Phage-encoded polysaccharide depolymerases in evolution

Due to the intimate co-evolution between phages and their bacterial hosts, polysaccharide diversity implies corresponding tail fiber and tailspike diversity. The highest level of diversity and mosaicism among phages within the operon of genes encoding structural proteins can be seen in the genes encoding receptor binding proteins (RBPs), reflecting the involvement of intensive vertical and horizontal transfer in the evolution of these proteins (Casjens and Molineux 2012). A common architecture of RBPs (including polysaccharide depolymerases) consists of three domains (Fig. 1). A small N-terminal domain is responsible for flexible connection of the RBP to the tail structure or to the baseplate. A large central domain forming the elongated homotrimeric structure is the key domain for host recognition and in the case of depolymerases possesses the enzymatic activity. The C-terminal domain seems to be responsible for protein trimerization and mainly works as an intra-molecular chaperone, while others also assign a function of receptor recognition to this domain (Weigele et al. 2003; Cornelissen et al. 2011; Schwarzer et al. 2012; Yan et al. 2014). The N-terminal as well as C-terminal domains of RBPs are conserved among phages belonging to the same group, while the host-specific central domain is highly variable and can be changed to modulate host range or to adapt to a new environment (Stummeyer et al. 2006; Schwarzer et al. 2012). Two different strategies of phages to adapt their RBPs to the polysaccharide receptor while conserving the same motif fold have been described: (1) changing the active site residues by mutation (vertical transfer) and (2) swapping with complete foreign domains specific for a different polysaccharide (horizontal transfer) (Barbirz et al. 2008; Leiman and Molineux 2008).

In the first strategy, many mutations are acceptable in the central domain as far as the stacking interactions between β-helix rungs remain intact (Leiman and Molineux 2008). This evolution can be observed between the homotrimeric tailspike of phage HK620, specific for *E. coli* O18A1, and similar tailspikes of phages P22 and Sf6 infecting *Salmonella* (Barbirz et al. 2008) (Table 1). Specifically, the active site residues involved are located on loops protruding from the β-helix structure, and thus, mutations therein do not affect the β-helix conformation. Two phages (HK620 and P22) do bind the polysaccharide between two protruding loops of the same monomer within the homotrimer (i.e., intra-subunit substrate binding), while in the case of phage Sf6, the polysaccharide binding site is located inter-subunit. These differences highlight the phage versatility to adjust the same protein fold to various substrates by mutation in different ways.

The second strategy is based on the exchange of complete tailspike modules to gain different specificity accordingly. This is clearly exemplified by the conserved N-terminal domain among the tailspikes of phages CUS-3, HK620, Sf6, and P22, while the catalytic domain of CUS-3 (i.e., an endosialidase, being specific for the *E. coli* K1 antigen) differs completely from the catalytic domain of the three others (Table 1). Moreover, the tailspike of phage K1F comprises the same catalytic domain as CUS-3 but shares the N-terminal domain of the T7 tail fiber (Stummeyer et al. 2006). These relationships clearly demonstrate evolution through horizontal transfer. A different, more complex configuration of tailspikes are the RBPs of podoviruses K1E, K1–5, and SP6. These viruses produce a specific adapter protein responsible for connection of the host-specific depolymerases to the virion. This adapter protein is equivalent in its function to the N-terminal domain of the tailspikes of CUS-3, HK620, Sf6, and P22 but is a larger, separate protein that provides the attachment site for two tailspikes, each with a different polysaccharide specificity. Tailspikes are interacting with the adapter protein by a short, conserved, N-terminal peptide (Leiman et al. 2007). Specifically, both phages K1E and K1–5 possess tailspikes with endosialidase activity specific for the K1 antigen. Phage K1–5 is equipped additionally with the K5 lyase enabling recognition of the K5 antigen (Scholl et al. 2001) (Table 1). Phage K1–5 has thus a dual specificity and can infect both K1 and K5 strains. Interestingly, the K1E has a small and probably truncated protein without enzymatic activity as the second tailspike and has therefore a more narrow host spectrum. Clarke et al. (2000) have proposed that phage K1E has evolved from phage K5A by the acquisition of the gene encoding the tailspike with endosialidase activity and the subsequent loss of the K5 lyase by a *kflA* gene truncation. *Salmonella* phage SP6 is morphologically and biologically similar to phages K1E and K1–5, but the catalytic domain of the tailspike is homologous to *Salmonella* phage P22 tailspikes with rhamnosidase activity involved in degradation of the *Salmonella* O-antigen, explaining the different host spectrum. At the same time, SP6 has a second but still unknown enzymatically active tailspike that broadens the host range to rough and smooth *Salmonella* strains, in contrary to phage P22 that infects only the smooth type (Scholl et al. 2002, 2004). *E. coli* bacteriophage Φ92 isolated in 1983 was reinvestigated by Schwarzer et al. (2012). Apart from the original host *E. coli* K92, which possesses a polysialic acid capsule, they found that this myovirus is also able to infect non-encapsulated *E. coli* laboratory strains such as EV5, a capsule defective derivative of a K1 strain; K-12 and B strains; and *Salmonella* strains (Kwiatkowski et al. 1983; Vimr and Troy 1985; Schwarzer et al. 2012) (Table 1). A cryoEM analysis of the Φ92 virion showed the presence of a multivalent host adsorption apparatus with at least five different receptor binding proteins being tailspikes or tail fibers. Moreover, the closely related phage PVP-SE1 encodes even six different tail fibers and spikes (Santos et al. 2011; Schwarzer et al. 2012). The best depolymerase-equipped phage is a recently described *Klebsiella* phage ФK64–1 (Pan et al. 2015, 2017) (Table 1). This giant virus was found to be able to propagate on a broad range of *Klebsiella* strains, with representatives of up to ten serotypes. ФK64–1 possess 11 genes encoding tail fibers, tailspikes, or lyases. Each expressed protein (S2–4, S1–1, S1–3, S2–2, S2–6, S2–3, S2–5, S1–2, S2–1) has activity specific to a different capsular polysaccharide (K1, K11, K21, K25, K30/K69, K35, K64, KN4, and KN5, respectively). Moreover, two proteins (S2–7 and S2–8) were not found active against any capsular serotype known until now (Pan et al. 2017). The electron micrographs of this giant phage revealed similar morphology to phage vB_KleM-RaK2 with broom-like tail fibers and several spike-like structures. Such high variety cannot be achieved in small bacteriophages belonging to *Podoviridae* family, which have a small tail, but is more likely in larger myoviruses.

### Methods for depolymerase activity determination

Determination of the depolymerizing activity is particularly challenging, as the difficulties to separate CPS/EPS or biofilm matrix from LPS complicate the analysis of polysaccharide degradation. As a consequence, the methods used so far are highly diverse but can generally be subdivided in biological, biochemical, and physical methods that can be either used generally independent of the polysaccharide structure and composition or used for specific substrates. The primary biological way to detect the presence of depolymerases is the application of spots of phages/enzyme on bacterial lawns and screening for the typical turbid halos (Cornelissen et al. 2011). Spectrophotometric methods allow a more quantitative determination of the depolymerase activity. For the evaluation of biofilm matrix degradation, a standard crystal violet or dimethyl methylene blue (DMMB) dye can be used to quantify biofilm matrix colorimetrically (Cornelissen et al. 2011, 2012; Tote et al. 2008). Spectrometric measurement of reducing sugars allows to determine the EPS/CPS digestion (Yurewicz et al. 1971; Rieger et al. 1975; Hsu et al. 2013). Other methods include examination of periodate oxidation of saccharides by determining the consumption of sodium periodate and the amount of formic acid formed (Kwiatkowski et al. 1983), evaluation of decrease of turbidity of insoluble EPS precipitated by cetylpyridinium chloride (CPC complexes) (Born et al. 2014; Majkowska-Skrobek et al. 2016), or uronic acid release (Glonti et al. 2010). Also, the crude separation of released sugars from polymer was analyzed by TLC chromatography (Glonti et al. 2010). Rheological analyses of alginate gels were done to measure the diffusion rate of phages through exopolysaccharides, as well as viscosity reduction measurements of treated polysaccharides (Thurow et al. 1974; Hanlon et al. 2001; Glonti et al. 2010). A couple of electrophoretic methods were also implemented to analyze depolymerase activity. The non-digested and digested polysaccharides can be compared on polyacrylamide gel electrophoresis (PAGE/SDS PAGE) stained by alcian blue/silver/methylene blue (Clarke et al. 2000; Barbirz et al. 2008; Majkowska-Skrobek et al. 2016). Fluorophore-assisted carbohydrate electrophoresis (FACE) (Chua et al. 1999) and capillary electrophoresis (Baker et al. 2002) are other electrophoretic methods that have been used. For more detailed analysis of the cleavage specificity, the lists of biochemistry techniques were implemented, such as ^1^H NMR spectroscopy, FABMS, MALDI MS, GC MS, ESI-MS, and EIMS (Thurow et al. 1974; Linnerborg et al. 2001). There is also the possibility to measure biofilm thickness by confocal laser scanning microscopy (CLSM) (Cerca et al. 2012), scanning electron microscopy (SEM), or atomic force microscopy (Chatterjee et al. 2014). A fully electric tuning fork resonance frequency measurement system designed by Piasecki et al. (2015) offered an effective mass measurement in a novel approach to evaluate the bacterial biofilm parameters such as mass, viscosity, and density. One of the more sophisticated techniques enabling precise measurement of biofilm matrix degradation is the laser interferometry detecting the small-molecule diffusion rate trough biofilm layer (Danis-Wlodarczyk et al. 2015, 2016).

### Applications of polysaccharide depolymerases

The unique ability of phage-derived depolymerases to specifically recognize and degrade CPS, EPS, and the O-antigen offers an attractive and promising tool for controlling pathogenic bacteria and other applications, including clinical diagnostics and biochemical analyses. Although most of these proteins do not seem to possess bactericidal activity as described for VALs and endolysins, they should be considered in therapy for their antivirulent potential (Majkowska-Skrobek et al. 2016). The loss or modification of bacterial surface structures that are used by many pathogens to promote virulence, host colonization, and biofilm formation makes bacteria less pathogenic and/or sensitizes them to either some antimicrobials or host defenses such as phagocytosis by macrophages and the bactericidal action of serum (Mushtaq et al. 2004, 2005; Zelmer et al. 2010; Bansal et al. 2014; Pan et al. 2015). Therapeutic efficacy of recombinant depolymerases that only modify the phenotype of the bacterial cells without affecting their viability and growth rate has been confirmed in animal models (Mushtaq et al. 2004; Waseh et al. 2010; Pan et al. 2015; Majkowska-Skrobek et al. 2016). When phage K1E endosialidase (endoNE) is administered intraperitoneally to neonatal rats infected with neuroinvasive *E. coli* K1 strains, a significant reduction of mortality due to bacteremia and the life-threatening systemic infection was observed (Mushtaq et al. 2004, 2005; Zelmer et al. 2010). In another study, the oral administration of *Salmonella*-specific endorhamnosidase from P22 phage (P22sTsp) to chickens prevented the pathogen’s colonization of the gut and its penetration into internal organs (Waseh et al. 2010). An interesting approach is the idea of alginate lyase application as an antibiofilm agent in combating *Pseudomonas aeruginosa* biofilm in the airways of cystic fibrosis patients (Glonti et al. 2010). Degradation of alginate, the exopolysaccharide produced by mucoid *P. aeruginosa* strains, can remove a barrier that protects bacterial cells from macrophages in the lungs, can perturb bacterial growth in biofilms, and decreases the viscosity of sputum (Hanlon et al. 2001; Glonti et al. 2010). Depolymerase-mediated biocontrol of pathogens has also been successfully attempted in a pathogen-plant system. Effective enzyme application has been observed in transgenic pears and apples that express the EPS-degrading enzyme from ΦEa1h phage, conferring resistance to fire blight caused by *Erwinia amylovora* (Malnoy et al. 2005; Flachowsky et al. 2008). An important challenge is the creation of depolymerases with lytic activity. A study carried out by Scholl et al. (2009) revealed that a recombinant fusion protein between the endorhamnosidase-active tailspike protein from *E. coli* O157-specific phage ΦV10 and a R-type pyocin is able to lyse *E. coli* O157:H7 strains and can be applied to eliminate the pathogen from food.

Phages able to express several depolymerases can also be constructed by synthetic biology (Lu and Collins 2007; Azeredo and Sutherland 2008). Potentially, in comparison to phages currently used in therapy, these genetically modified phages with a broader range of activities would be more effective or even target other serotypes. Depolymerases could also be applied in combination with conventional antimicrobials against multidrug-resistant pathogens, notably those living in biofilm communities. The synergistic action of either phages producing depolymerase or recombinant depolymerase with conventional chemical agents, including antibiotics and disinfectants, have already been shown to suppress *Klebsiella* infections (Verma et al. 2009, 2010; Bansal et al. 2014; Chai et al. 2014). Moreover, the ability of phages to express not only their own but also other bacterial enzymes indicates their potential to specifically target pathogens possessing for example chondroitin capsules (Cress et al. 2014). Besides combating bacterial infections, depolymerases can be used as an alternative to antiserum for the typing of bacterial strains and the detection of polysaccharides in immunohistological studies. The first test of this concept was initially realized with *Klebsiella* strains. Owing to the high specificity of depolymerases, it was suggested that enzyme-based capsular typing may be more useful than whole phage typing (Hsu et al. 2013; Lin et al. 2014). The analogous approach has been successfully applied to develop an endosialidase-based detection reagent for identification of polysialic acid-containing bacteria as well as eukaryotic cells (Jakobsson et al. 2015). As a detection tool, these enzymes are used in a catalytically inactive form, capable to recognize and bind the substrate, but without its degradation. Their potential advantages that give them the supremacy over antibodies are (i) the production based on recombinant technology without the use of animals, (ii) the lack of cross-reactivity with cell surface proteins, and (iii) the lack of toxicity. It is also worth noting that endosialidases, besides therapeutic and diagnostic significance, are also of interest as tool to degrade artificial polysialic acid-based hydrogels and other derivatives used as a scaffold biomaterial in neurobiology (Berski et al. 2008).

## Virion-associated lysins

VALs are generally linked to a component of the virion and therefore are also known as VAPGHs (Rodríguez-Rubio et al. 2013a), tail-associated muralytic enzymes (TAMEs) (Paul et al. 2011), and structural lysins or exolysins (Oliveira et al. 2013). However, all these names are confusing. First of all, some VALs are in fact lytic transglycosylases, which are not hydrolases. Secondly, the phage tail is the most common attachment site for VALs, but not the only one. VAL can also be anchored to the phage neck, baseplate, or can be embedded in the viral membrane. Thirdly, some VALs do not serve as structural elements but are connected to the genomic phage DNA or occur as free form enclosed in the capsid (Table 2). Finally, exolysins are generally associated with enzymes secreted by a bacterium to kill competing bacteria. For these reasons, virion-associated lysin is the most appropriate name and is used in this review.

**Table 2:** Phage-encoded, virion-associated lysins

Phage phylogeny	Phage	Protein name/accession code	Location in the virion	Monomer mass (kDa)/oligomeric state (if known)	Putative or experimentally confirmed activity*	Phage host	References
Gram negative-infecting phages							
*Podoviridae*	T3	Gp16 NP_523341.1	Internal head protein that forms part of extensible tail	143/trimer	Lytic transglycosylase	*Escherichia coli*	Moak and Molineux (2004) and Serwer et al. (2014)
	T7	Gp16 NP_042004.1	Internal head protein that forms part of extensible tail	143/trimer	Lytic transglycosylase	*Escherichia coli*	Moak and Molineux (2000, 2004), Molineux (2001), and Serwer et al. (2008)
	ΦYeO3–12	Gp16 NP_052116.1	Putative internal head protein that forms part of extensible tail	143	Lytic transglycosylase	*Yersinia enterocolitica*	Moak and Molineux (2004)
	SP6	Gp36 NP_853596.1	Internal head protein that forms part of extensible tail	107	Lysozyme	*Salmonella typhimurium*	Moak and Molineux (2004)
	K1–5	Gp38 YP_654136.1	Unknown	109	Lysozyme	*Escherichia coli*	Moak and Molineux (2004)
	ΦKMV	Gp36 NP_877475.1	Putative internal head protein that forms part of extensible tail. Part of the phage injection needle	115	Lysozyme	*Pseudomonas aeruginosa*	Briers et al. (2006) and Lavigne et al. (2004)
	P22	Gp4 NP_059632.1	Phage neck (head-tail connector)	18	Lysozyme	*Salmonella typhimurium*	Moak and Molineux (2004) and Tang et al. (2011)
*Siphoviridae*	T5	Pb2 YP_006968.1	Tail tip (forms tail tube)	124/multimeric protein	Muralytic	*Escherichia coli*	Boulanger et al. (2008), Moak and Molineux (2004), and Zivanovic et al. (2014)
*Myoviridae*	T4	Gp5 NP_049757.1	Cell-puncturing device on phage baseplate	63/homotrimer	Lysozyme*	*Escherichia coli*	Arisaka et al. (2003), Kanamaru et al. (2002), and Nishima et al. (2011)
	ΦKZ	Gp181 NP_803747.1	Needle-like cell-puncturing device	246/putative trimer	Glycosidase (lysozyme or lytic transglycosylase)	*Pseudomonas aeruginosa*	Briers et al. (2008)
*Corticoviridae*	PM2	P7 NP_049902.1	Lipid core-associated, integral membrane protein	37	Muralytic	*Pseudoaltero-monas* sp.	Kivelä et al. (2004)
*Tectiviridae*	PRD1	P7 NP_040700.1 P15 NP_040683.1	Viral membrane-associated protein (responsible for infection)	27 17	Lytic transglycosylase Lytic transglycosylase	*Escherichia coli*	Rydman and Bamford (2000, 2002)
*Cystoviridae* (dsRNA)	Φ6	P5 NP_620343.1	Exposed on the outside of the nucleocapsid surface	24/monomer	Endopeptidase*	*Pseudomonas savastanoi* pv. *phaseolicola*, *Pseudomonas syringae* pv. *phaseolicola*	Caldentey and Bamford (1992) and Mindich and Lehman (1979)
Gram positive-infecting phages							
*Podoviridae*	ΦP68	P17 NP_817333.1	Virion-associated	75	Muralytic	*Staphylococcus aureus*	Takác and Bläsi (2005)
	Φ29	Gp3 YP_002004530.1	Associated to DNA and free form packed in capsid	31	Lysozyme	*Bacillus subtilis*	Moak and Molineux (2004)
	GA-1	P11 (Gp3) NP_073686.1	Probably associated to DNA	30	Lysozyme	*Bacillus* G1R	Moak and Molineux (2004)
	M2	Gp3 (unknown)	Probably associated to DNA	31	Lysozyme	*Bacillus subtilis*	Moak and Molineux (2004)
*Siphoviridae*	SP-β prophage	CwlP (YomI) NP_046584.1	Probably tail	252	Lysozyme and endopeptidase*	*Bacillus subtilis*	Sudiarta et al. (2010)
	Tuc2009	Tal_2009_ NP_108727.1	End of the phage tail (tail tip)	101/trimer	Endopeptidase*	*Lactococcus lactis*	Kenny et al. (2004), McGrath et al. (2006), and Stockdale et al. (2013)
	TP901–1	Tal_901–1_ NP_112710.1	End of the phage tail (tail tip)	102/trimer	Endopeptidase	*Lactococcus lactis*	Stockdale et al. (2013)
	ΦMR11	Gp61 YP_001604152	End of the phage tail (tail tip)	71	Amidase (N-term) and lysozyme (C-term)	*Staphylococcus aureus*	Rashel et al. (2008)
	ΦIPLA88	HydH5 YP_002332533.1	Baseplate (probably)	72	Amidase (N-term) and lysozyme (C-term)	*Staphylococcus aureus*	Rodríguez et al. (2011) and Rodríguez-Rubio et al. (2012)
	Φ11	Gp49 YP_001604152.1 Gp44 (Tal) NP_803297.1	Baseplate (probably) Baseplate (probably)	72 71	Lysozyme and amidase Endopeptidase (N-term) and lipase or esterase (C-term)	*Staphylococcus aureus*	Moak and Molineux (2004), Li et al. (2016), and Rodríguez-Rubio et al. (2013c)
*Myoviridae*	K	ORF56 AAO47505.1	Tail	91	Endopeptidase	*Staphylococcus* sp.	Paul et al. (2011)

### Enzymatic mechanism of action and specificity of VALs

Based on the type of the chemical bond that is cleaved, the specificity of VALs is classified into three categories: (i) glycosidases, further subdivided into lysozymes, glucosaminidases, and lytic transglycosylases, cleaving one of the two glycosidic bonds in the glycan chain; (ii) amidases cutting the amide bond between the lactyl group of N-acetylmuramic acid and L-alanine of the stem peptide; and (iii) endopeptidases cleaving within the stem peptide or the cross-link (Young 1992). The vast majority of described VALs, including the best studied examples from coliphages T4 (Gp5—lysozyme) and T7 (Gp16—lytic transglycosylase) (Yap and Rossmann 2014; Moak and Molineux 2000) are glycosidases (Table 2), and technically, only those enzymes are carbohydrate active enzymes. Lysozymes and lytic transglycosylases cleave the same β-1,4-glycosidic linkage between N-acetylmuramic acid and N-acetyl-D-glucosamine. However, lytic transglycosylases are not hydrolases like lysozymes as they do not use a water molecule during cleavage but instead catalyze the concomitant formation of a 1,6-anydromuramoyl product (Scheurwater et al. 2008). Endopeptidase activity is generally attributed to VALs of Gram-positive-specific phages such as ORF56, Gp44, Tal(2009), and Tal(901–1) and CwlP encoded by phages K, Φ11, Tuc2009, TP901–1, and SP-β, respectively (Sudiarta et al. 2010; Paul et al. 2011; Stockdale et al. 2013) (Table 2). Only one VAL originating from a Gram-negative-infecting phage, specifically Gp5 of *Pseudomonas* phage Φ6 (dsRNA, *Cystoviridae*), has been demonstrated to possess endopeptidase activity (Caldentey and Bamford 1992). VALs with amidase activity include Gp61, HydH5, and Gp49 of *Staphylococcus*-specific *Siphoviridae* ΦMR11, ΦIPLA88, and Φ11 (Rashel et al. 2008; Rodríguez et al. 2011) (Table 2).

### Various architectures of VALs

In terms of structure, VALs are highly diverse enzymes. Differences relate to the number of domains and their organization, molecular weight, and oligomeric state. The structure of VALs is modular and reminiscent of the structure of many polysaccharide depolymerases. It consists of a domain that binds to the virion and one or two lytic domains responsible for PG degradation. An additional domain, responsible for cell wall binding, was found in P17 VAL of staphylococcal phage P68 (Takác and Bläsi 2005). The majority of VALs possess a single lytic domain. Only five VALs with two different lytic domains have been reported for *Staphylococcus* phages (Table 2), which are CwlP of prophage SP-β with lysozyme and endopeptidase domains (Sudiarta et al. 2010), Gp44 of phage Φ11 with endopeptidase and SGNH hydrolase domains (Li et al. 2016), Gp61 of phage ΦMR11 (Rashel et al. 2008), HydH5 of phage ΦIPLA88 (Rodríguez et al. 2011; Rodríguez-Rubio et al. 2012, 2013b), and Gp49 of phage Φ11 (Rodríguez-Rubio et al. 2013c; Li et al. 2016), all with lysozyme and amidase activities. Interestingly, all VALs with two lytic domains are encoded by phages specific for Gram-positive bacteria, which may be related with the thick cell wall of Gram-positive cells (Table 2). The order of VAL domains is also variable. Lytic active domains can be located at the N-terminus (e.g., P17 of phage P68, Gp3 of phage Φ29) (Moak and Molineux 2004; Takác and Bläsi 2005), at the C-terminus (e.g., Gp36 of phage ΦKMV and Pb2 of phage T5) (Lavigne et al. 2004; Boulanger et al. 2008), or at both termini of the protein (e.g., Gp44 and Gp49 of phage Φ11) (Li et al. 2016). Equally large diversity is observed for the molecular weight of VALs within the range of 37–252 kDa, with the smallest being the internal membrane VAL P7 of phage PM2 (Kivelä et al. 2004) and the largest being CwlP of prophage SP-β (Sudiarta et al. 2010), respectively. The oligomeric state has only been characterized for a few VALs. They can be present as monomers like the viral membrane-associated Gp5 of phage Φ6 (Caldentey and Bamford 1992) but also as a dodecamer like Gp4 found in neck of phage P22 (Tang et al. 2011). However, a trimeric state as found in Gp5, component of the hub of the T4 phage baseplate (Kanamaru et al. 2002), Tal(2009) and Tal(901–1) forming the tail tips of phages Tuc2009 and TP901–1, respectively (Stockdale et al. 2013), is the most frequently described oligomeric state. It is also worth to mention that *E. coli* phage PDR1 (*Tectiviridae*) and *Staphylococcus* phage Φ11 (*Siphoviridae*) are the only known examples of bacterial viruses equipped with two different VALs (Table 2).

### Location of VALs in the virion

VALs appear in various parts of the virion. In the case of *Tectiviridae*, membrane-containing coliphages like PRD1 (proteins P7 and P15) and Φ6 (Gp5) VALs are associated with the viral membrane and exposed on the outside of the nucleocapsid surface (Caldentey and Bamford 1992; Rydman and Bamford 2000, 2002). The phage tail is the most common location of VALs encoded by *Caudovirales*, wherein the tail tip is the most typical location of VALs from *Siphoviridae* attacking Gram positives (Table 2). Another location is the baseplate where VAL oligomers form a cell-puncturing device. Baseplate location is characteristic for Gp5 of T4 coliphage (Kanamaru et al. 2002) and gp181 of *Pseudomonas* phage ΦKZ (Briers et al. 2008; Fokine et al. 2008). The Gp4 of the *Salmonella*-specific phage P22 is the only VAL described so far that is located at the phage neck (Tang et al. 2011). The Gp16 encoded by phages T7, T3, and øYeO3–12 and Gp36 of phage SP6 are internal head proteins that form part of an extensible tail, which is characteristic for most Gram-negative-specific *Podoviridae* (Struthers-Schlinke et al. 2000; Moak and Molineux 2004; Serwer et al. 2008, 2014). Also, Gp3 of phage Φ29 and probably Gp3 of the similar phages GA-1 and M2 as well are located in the phage head but are linked to the genomic DNA. VALs of these *Podoviridae* infecting Gram-positive *Bacillus* sp. are ejected from the virion prior the DNA, allowing efficient infection (Moak and Molineux 2004). Moreover, it was demonstrated that a small portion of the free form of Gp3 (not attached to DNA) is enclosed inside the capsid of phage Φ29 and may move down to the tail tip, digesting the peptidoglycan together with the portion of Gp3 that is covalently bound to DNA (Moak and Molineux 2004).

### Methods for activity determination of VALs

As for depolymerases, there is no standard procedure for the evaluation of the enzymatic activity of VALs. However, the variability in peptidoglycan composition is lower than the one of EPS, CPS, and O-polysaccharide, resulting in some more common practices for which either purified substrates are used (PG, sugar chains, peptides) or whole cells. These cells can be either a living or a dead, autoclaved cell suspension. In case of Gram-negative cells, the outer membrane has to be removed first to make the PG accessible. Outer membrane permeabilization is hereby achieved by chloroform vapor that dissolves the lipid inner and outer membranes. The lytic activity of most described VALs has been demonstrated on purified PG using in-gel activity staining (zymography) or by spectrophotometry using whole cells (Caldentey and Bamford 1992; Kaberdin and McDowall 2003﻿; ﻿Moak and Molineux 2004; Kivelä et al. 2004; Lavigne et al. 2004; Sudiarta et al. 2010; Briers et al. 2006, 2008; Rodríguez et al. 2011; Stockdale et al. 2013; Rodríguez-Rubio et al. 2012). Both methods imply the analysis of the reduction in turbidity of the substrate, either as transparent bands in turbid gels (zymography) or by spectrophotometry measuring the optical density. The spectrophotometric approach is the most accurate to quantify the enzymatic (U/ml) or specific (U/mg) activity. It is also commonly used for endolysin testing and relies on determination of the rate of reduction of the optical density of a substrate suspension at 600 nm upon addition of a given amount of enzyme (expressed as ΔOD_600_/μg min) (Donovan et al. 2006; Donovan and Foster-Frey 2008; Becker et al. 2009; Rodríguez-Rubio et al. 2012). The general limitation of this method is the high “day-to-day” variability in the results (Donovan and Foster-Frey 2008). A standardized approach for enzymatic activity determination of PG-degrading endolysins against Gram-negative strains has been proposed by Briers and co-workers (Briers et al. 2007a) and relies on the determination of the linear region of the turbidity drop. Enzymatic activity is expressed in U, often corresponding to a linear descent in OD_600_ of 0.001 per min (Cheng et al. 1994). This method has been used for determination of enzymatic activity of VALs of phages ΦKMV (Lavigne et al. 2004) and ΦKZ (Briers et al. 2008). The cleavage specificity of phages lysins is only rarely experimentally examined and is often based on in silico homology and therefore biased by historic misannotations. However, in those few experimentally validated cases, liquid chromatography–mass spectrometry is the most common method (Caldentey and Bamford 1992; Sudiarta et al. 2010; Stockdale et al. 2013; Rodríguez-Rubio et al. 2016; Maciejewska et al. 2017).

### Use of lysins as antibacterial proteins

VALs possess properties that make them potential weapons to prevent and combat bacterial pathogens. Indeed, purified VALs kill Gram-positive cells when they are added exogenously as the PG is directly accessible and its breakdown results in osmotic lysis and immediate cell death. Similar to the use of endolysins for the same goal, VALs are featured by (i) high specificity, targeting only a subset of pathogens; (ii) desirable mode of action on unique and highly conserved bonds in PG, reducing the risk on fast emergence and spread of resistance; (iii) lytic and antibacterial activities regardless of the presence of antibiotic resistance mechanisms; and (iv) additional useful features like thermostability, high ionic strength tolerance, and synergistic activity with antibiotics or endolysins. The antibacterial activity of VALs against Gram-positive bacteria mainly focused on *Staphylococcus aureus.* The P17 VAL was shown to have a spectrum activity against most of *S. aureus* clinical strains, including isolates resistant to phage P68, the producer of P17 (Takác and Bläsi 2005). Antistaphylococcal activity was also demonstrated for both lytic domains of Gp61 of phage ΦMR11, causing lysis of approximately 80% of *S. aureus* cells within 30 min (Rashel et al. 2008). Other VALs lethal to *S. aureus* are ORF56 of phage K (Paul et al. 2011) and HydH5 of phage ΦIPLA88, which were shown to lyse *S. aureus* (including methicillin-resistant *S. aureus* (MRSA)) and *S. epidermidis*. Moreover, HydH5 is thermostable up to 100 °C for 5 min and acts synergistically with endolysin LysH5 (Rodríguez et al. 2011; Rodríguez-Rubio et al. 2012). Regarding Gram-negative pathogens, VALs have been demonstrated to lyse the peptidoglycan of outer membrane-permeabilized cells. Gp36 of phage ΦKMV and Gp181 of phage ΦKZ showed lytic activity against *P. aeruginosa* with a specific activity of 4800 U/mg (similar to commercial hen egg white lysozyme) and 60,000 U/mg, respectively (Lavigne et al. 2004; Briers et al. 2006, 2008). Furthermore, Gp36 possesses a highly thermostable, C-terminal lysozyme domain retaining activity after treatments of at 95 °C, whereas the Gp181 tolerates high ionic stress (>320 mM) (Briers et al. 2008). Gp5 of *Pseudomonas* phage Φ6 is lytic in vitro against several outer membrane-permeabilized, Gram-negative species (*Pseudomonas phaseolicola*, *P. aeruginosa*, *P. fluorescens*, *P. putida*, *E. coli*, *S. typhimurium*, and *Proteus vulgaris*) (Caldentey and Bamford 1992). In spite of a natural exogenously acting function, lysins can generally not penetrate the outer membrane when not associated with the virion. Therefore, recombinant lysins can only kill intact Gram-negative bacteria in presence of outer membrane permeabilizers such as EDTA. Genetic modification with outer membrane-permeabilizing peptides as has been described for endolysins in the development of artilysins (Briers et al. 2014; Gerstmans et al. 2016) can be an option. In context of the improvement of the potential of VALs as antibacterials, different chimeric proteins comprising one or more domains of lysins have been constructed and characterized. P16–17 comprising the N-terminal endopeptidase domain of the endolysin (P16) and the C-terminal cell wall binding domain of the VAL (P17) of *Staphylococcus* phage 68 shows a high and specific antibacterial activity against *S. aureus* strains. P16–17 acts synergistically with gentamicin and increases bacterial sensitivity towards this drug (Manoharadas et al. 2009). Protein P128, a chimera of the N-terminal endopeptidase of the structural lysin from staphylococcal K phage and the cell wall-binding SH3 of lysostaphin, was demonstrated to possess strong antistaphylococcal activity and was successfully used in decolonization of rat nares from staphylococcal infection, reducing the bacterial load by two orders of magnitude after treatment. Moreover, P128-resistant mutants were shown to lose the β-lactam drug resistance phenotype and become hypersensitive to β-lactams (Paul et al. 2011; Saravanan et al. 2013; Sundarrajan et al. 2014). Two other chimeric proteins, i.e., HydH5Lyso and HydH5SH3b, were prepared based on the HydH5 from *S. aureus*-specific phage ΦIPLA88. The HydH5Lyso (a fusion of HydH5 with lysostaphin) and HydH5SH3b (fusion of HydH5 with lysostaphin binding domain SH3b) were able to lyse MRSA and *S. epidermidis* cells with higher activity than each of the enzymes separately. Both chimeric proteins have proven their usefulness as effective milk biopreservatives (Rodríguez-Rubio et al. 2012). Another chimeric protein composed of the endopeptidase domain of the VAL and the cell wall binding domain of the endolysin of *Enterococcus faecalis* phage F170/08 is the EC300 protein. The muralytic activity of EC300 showed enhanced activity over the original lysins against clinical, multidrug resistant *E. faecalis* strains (Proença et al. 2015).

In conclusion, phages have evolved elaborate, virion-associated carbohydrate active enzymes that accommodate the existing diversity in carbohydrate barriers presented to phages during infection. This intimate co-evolution with a high evolutionary rate has provided us with a highly versatile tool kit of specific proteins to either kill, sensitize, or detect bacteria.
